# Impact of Tumor Location on Aesthetic Outcomes in Oncoplastic Breast-Conserving Surgery: A Retrospective Comparison of Three Acellular Dermal Matrix Techniques

**DOI:** 10.3390/cancers17081293

**Published:** 2025-04-11

**Authors:** Hyung-Suk Yi, Han Gwak, Jin-Hyung Park, Sung-Ui Jung, Jin-Hyuk Choi, Ku-Sang Kim, Yoon-Soo Kim

**Affiliations:** 1 Department of Plastic and Reconstructive Surgery, Kosin University Gospel Hospital, Kosin University College of Medicine, Busan 49267, Republic of Korea; sencha21@naver.com (H.-S.Y.); omerta222@naver.com (H.G.); atreyue@naver.com (J.-H.P.); 2 Department of Surgery, Kosin University Gospel Hospital, Kosin University College of Medicine, Busan 49267, Republic of Korea; ist2000good@daum.net (S.-U.J.); drchoijinhyuk@gmail.com (J.-H.C.); ideakims@gmail.com (K.-S.K.)

**Keywords:** oncoplastic breast surgery, breast-conserving surgery, acellular dermal matrix, tumor location, aesthetic outcomes, patient satisfaction, breast reconstruction

## Abstract

**Background/Objectives**: Oncoplastic breast-conserving surgery (OBCS) with acellular dermal matrix (ADM) has gained prominence in contemporary breast cancer management, yet evidence-based guidelines for selecting optimal ADM techniques based on the tumor location remain undefined. This investigation evaluated the impact of the tumor quadrant location and ADM application methodology on aesthetic outcomes, patient satisfaction, and postoperative complications following OBCS. **Methods**: This retrospective comparative analysis examined 229 patients who underwent OBCS with immediate ADM-based volume replacement (2020–2022) utilizing three distinct techniques: the diced ADM with sheet technique (n = 102), the diced ADM with paste-type micronized technique (n = 70), or the diced ADM-only technique (n = 57). The outcomes were stratified by tumor location (superomedial, superolateral, inferomedial, and inferolateral) and evaluated using validated physician-assessed aesthetic scores (four-point scale), patient satisfaction metrics, and comprehensive complication profiling with 24-month minimum follow-up. **Results**: The tumor quadrant location significantly influenced aesthetic outcomes across all techniques (*p* < 0.001), with superolateral quadrants demonstrating superior results (3.5 ± 0.4). In the superomedial quadrant, the diced ADM with paste-type micronized technique yielded significantly higher aesthetic scores than the diced ADM-only technique (3.2 ± 0.5 vs. 2.8 ± 0.6, *p* = 0.032, Cohen’s d = 0.71). In the superolateral quadrant, the diced ADM-only technique achieved superior outcomes compared with the diced ADM with sheet technique (3.6 ± 0.4 vs. 3.4 ± 0.5, *p* = 0.020, d = 0.44). For inferomedial defects, the diced ADM with sheet technique demonstrated significant advantages over the diced ADM-only technique (2.9 ± 0.7 vs. 2.7 ± 0.7, *p* = 0.005, d = 0.29). A consistent discrepancy between physician and patient assessments was observed, particularly in medial quadrant reconstructions (mean difference: 0.2 points, *p* = 0.003). **Conclusions**: The optimal ADM technique varied significantly by tumor location. The diced ADM with paste-type micronized technique demonstrated superior outcomes in superomedial defects, the diced ADM with sheet technique provided essential structural support for inferomedial reconstructions, and the diced ADM-only technique achieved excellent results with reduced material requirements in superolateral locations. These findings provide preliminary evidence that may guide quadrant-specific ADM technique selection in OBCS, potentially enhancing aesthetic outcomes, patient satisfaction, and resource utilization.

## Simple Summary

Oncoplastic breast-conserving surgery aims to remove tumors while preserving aesthetics, but outcomes vary depending on the tumor location. This study investigated how three different acellular dermal matrix techniques performed across different breast quadrants. We found that specific techniques worked better in particular locations: a paste-type matrix enhanced the results for upper inner tumors, sheet reinforcement benefited lower inner tumors, and a diced matrix alone achieved excellent results for upper outer tumors. These location-specific recommendations can help surgeons select optimal reconstruction approaches, potentially improving breast appearance, patient satisfaction, and resource utilization while maintaining oncologic safety. Our findings provide preliminary evidence that may guide surgeon decision-making based on the tumor location.

## 1. Introduction

Oncoplastic breast-conserving surgery (OBCS) represents a significant advancement in breast cancer management, effectively integrating oncologic tumor resection with sophisticated plastic surgery techniques to achieve both complete tumor removal and optimal aesthetic outcomes [1,2]. This approach has transformed the surgical paradigm by preserving breast shape and symmetry, which positively impacts patient body image and quality of life [3,4]. Despite these advantages, aesthetic outcomes following OBCS can be significantly influenced by several critical factors. Previous meta-analyses and clinical studies have consistently identified the tumor-to-breast volume ratio and the tumor location as primary determinants of cosmetic outcomes [5,6]. Recent work by Di Leone et al. (2022) has further demonstrated that sophisticated level II oncoplastic techniques can serve as effective alternatives to mastectomy with reconstruction, even in challenging clinical scenarios [7]. Notably, tumors situated in the medial or inferior breast quadrants have been associated with higher rates of postoperative deformities and inferior aesthetic results after conventional lumpectomy compared with other quadrants [8,9].

The application of acellular dermal matrix (ADM) in breast reconstruction has evolved significantly since its initial introduction in the early 2000s. First described for use in implant-based total breast reconstruction by Breuing and Warren in 2005, ADM was subsequently adapted for partial breast reconstruction following breast-conserving surgery. By 2010, several groups had reported preliminary experiences with ADM in volume replacement techniques for partial mastectomy defects. The technique has undergone continuous refinement over the past decade, with innovations in ADM preparation methods, application techniques, and surgical approaches [10,11]. Our previous work (Kim et al., 2022) investigated the correlation between ADM volume and implant size selection, providing valuable guidance for material optimization in direct-to-implant reconstruction [12]. The current study builds upon this established foundation, specifically examining the impacts of different ADM formulations and application methods in the context of tumor location-specific reconstruction.

ADM has emerged as an increasingly valuable tool in OBCS for volume replacement due to its biocompatibility, ready availability, and avoidance of donor site morbidity [13,14]. ADM functions as a biologic scaffold for tissue regeneration, promoting cellular infiltration, angiogenesis, and integration with surrounding tissues at the surgical site [15]. Several configurations of ADM are currently utilized in partial breast reconstruction, including diced ADM, sheet ADM, and paste-type (micronized) ADM, each with distinct physical properties that potentially influence reconstructive outcomes [16]. Sheet ADM provides structural support and defined borders, while paste-type formulations offer enhanced moldability for complex contours, and diced ADM combines aspects of both with intermediate handling properties [17].

Previous studies have demonstrated the overall safety and efficacy of various ADM applications in breast reconstruction. Gwak et al. reported that filling lumpectomy cavities exclusively with diced ADM is safe and achieves high rates of patient cosmetic satisfaction in selected cases [18]. Similarly, An et al. found that particulate (pellet-type) ADM can recreate breast contours more effectively than sheet forms alone, with high patient satisfaction and reduced complication rates [19]. Park et al. demonstrated favorable outcomes using sheet-type ADM for partial breast reconstruction across various defect types [20]. However, none of these studies provided specific recommendations for which ADM technique is optimal for a given tumor location within the breast. The unique anatomical challenges presented by each breast quadrant—including variations in tissue thickness, vascular supply, gravitational effects, and proximity to the nipple–areola complex—likely influence reconstruction outcomes significantly [21,22], yet evidence-based guidance for quadrant-specific ADM selection has been notably absent from the literature.

Recent advancements in ADM applications for breast reconstruction have further expanded the technical options available to oncoplastic surgeons. Sbitany et al. (2020) described a novel technique for ADM implantation and fixation in prepectoral reconstruction, demonstrating excellent aesthetic outcomes with reduced animation deformity [23]. Turgeon et al. (2024) reported on the clover technique for oncoplastic reconstruction in small- to medium-sized breasts, expanding the arsenal of approaches available to surgeons [24]. Additionally, de Vita et al. (2014) reported on the long-term safety and effectiveness of combined breast techniques under local anesthesia, highlighting the evolving scope of minimally invasive approaches in breast surgery [25]. These recent innovations underscore the dynamic nature of the field and the continued refinement of ADM-based techniques. Despite these advancements in surgical techniques, standardized protocols for oncoplastic breast-conserving procedures continue to evolve [26], with emerging long-term outcome data supporting the oncologic safety and aesthetic durability of properly executed oncoplastic approaches [27].

Our study addresses this knowledge gap by systematically comparing three distinct ADM application techniques across four breast quadrants, offering initial insights that may inform tumor location-specific ADM selection in OBCS. We hypothesized that the optimal ADM-based oncoplastic technique differed according to the tumor location within the breast due to the unique anatomical and biomechanical challenges presented by each quadrant. This investigation aimed to (1) evaluate aesthetic outcomes and patient satisfaction across different breast quadrants using three ADM volume replacement techniques, (2) analyze complication rates for each technique in each quadrant, and (3) compare physician assessments with patient-reported satisfaction. The clinical implications of this research are significant, potentially enhancing surgical decision-making, improving aesthetic outcomes, reducing complication rates, and enabling more precise preoperative patient counseling regarding the expected results based on the tumor location.

By providing initial insights for quadrant-specific technical guidance for ADM application in OBCS, this study represents an important step toward optimizing oncoplastic approaches for breast cancer patients, ultimately improving both oncologic and aesthetic outcomes across diverse clinical scenarios.

## 2. Materials and Methods

### 2.1. Study Design and Patient Population

This retrospective comparative analysis evaluated the impacts of tumor location and acellular dermal matrix (ADM) application technique on aesthetic outcomes, patient satisfaction, and complication rates following oncoplastic breast-conserving surgery (OBCS). The study cohort comprised 229 female patients who underwent partial mastectomy with immediate ADM-based volume replacement at Kosin University Gospel Hospital between January 2020 and December 2022.

The inclusion criteria were rigorously defined as (1) histologically confirmed primary breast cancer (ductal carcinoma in situ or invasive carcinoma) treated with breast-conserving surgery; (2) immediate volume replacement reconstruction utilizing ADM; and (3) availability of comprehensive clinical documentation, including standardized preoperative and postoperative photographs and patient-reported outcome measures, with a minimum follow-up duration of 24 months.

**Figure 1:**
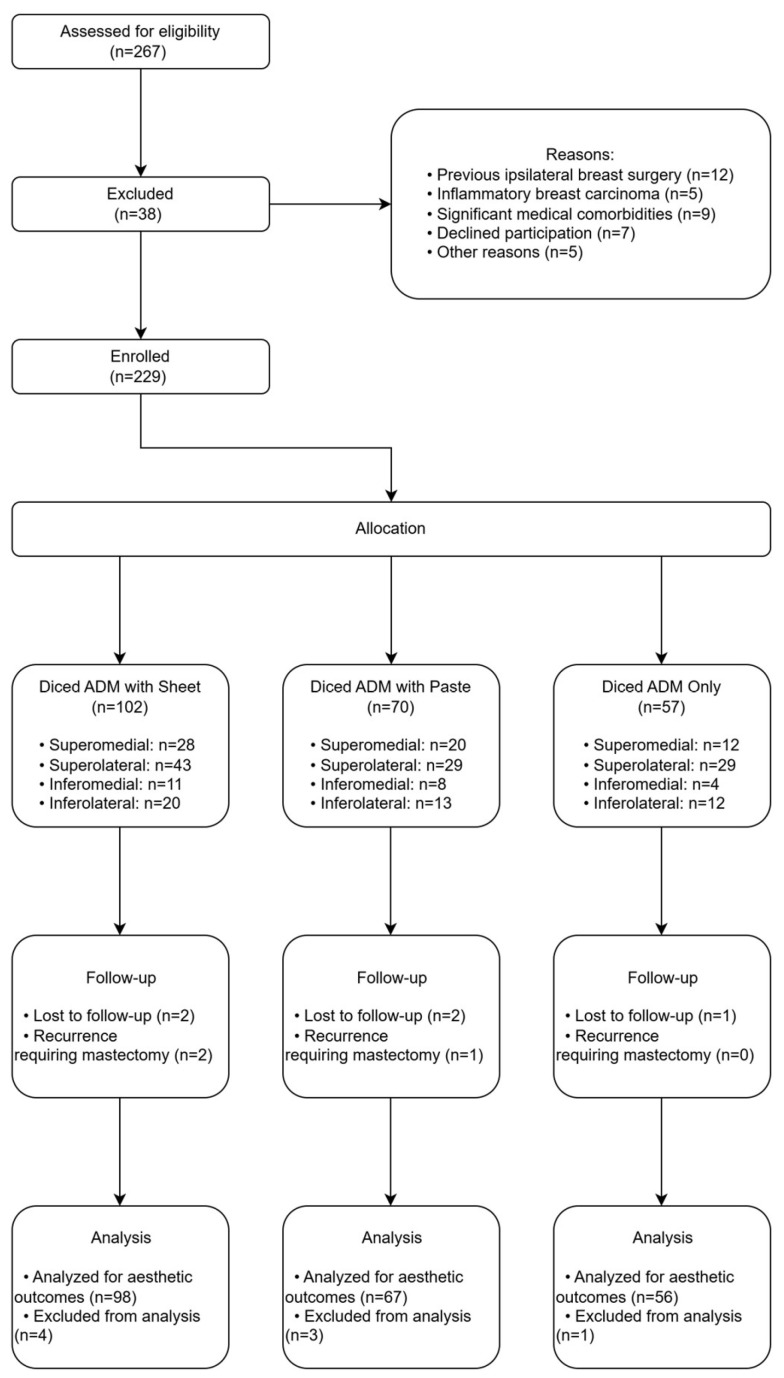
CONSORT diagram illustrating the patient selection and allocation process. The initial screening identified 267 potential candidates, with 229 patients meeting inclusion criteria and proceeding to oncoplastic breast-conserving surgery with ADM reconstruction. Patients were allocated to three ADM techniques based on preoperative assessment criteria, with distribution across all four breast quadrants. Five patients were lost to follow-up during the 24-month minimum follow-up period, and three patients who developed local recurrence requiring mastectomy were excluded from aesthetic outcome analysis.

The exclusion criteria encompassed (1) previous ipsilateral breast surgical intervention (excluding diagnostic biopsies), (2) inflammatory breast carcinoma, and (3) significant medical comorbidities (e.g., uncontrolled diabetes mellitus or active connective tissue disease) with potential to adversely affect wound healing or aesthetic outcomes. The study protocol was designed in accordance with the ethical principles delineated in the Declaration of Helsinki and received approval from the Institutional Review Board of Kosin University Gospel Hospital (IRB No. 2024-02-015). Written informed consent was obtained from all patients prior to their inclusion in the study (Figure 1).

### 2.2. ADM Terminology and Definitions

For clarity, we define the following ADM formulations used in this study:

- Sheet ADM: A continuous, flat piece of acellular dermal matrix with defined borders that provides structural support and serves as a scaffold for tissue integration.
- Diced ADM: Small fragments (approximately 1–2 mm^3^) of acellular dermal matrix that can conform to irregular spaces and provide volume replacement without rigid structural properties.
- Paste-type (micronized) ADM: Ultra-small particulate acellular dermal matrix combined with sterile saline to create an injectable substance with high moldability for precise contour refinement.

### 2.3. Data Collection

Comprehensive data collection was conducted through a systematic review of electronic medical records and a prospectively maintained oncoplastic breast surgery database. Two independent investigators (H.K. and H.S.Y.) extracted data using a standardized collection instrument, with discrepancies resolved through consensus discussion. The following variables were meticulously documented for each patient:

- Demographics: Age and body mass index (BMI).
- Tumor characteristics: Quadrant location, laterality, histological classification, tumor grade, and pathological dimensions.
- Surgical parameters: Date of procedure, ADM technique employed, volumetric quantification of ADM utilized, incision pattern, and axillary staging procedure.
- Adjuvant therapy: Chemotherapy and radiotherapy administration.
- Aesthetic outcomes: Panel-based physician aesthetic evaluation scores and patient satisfaction metrics.
- Postoperative complications: Including depression deformity, bulging deformity, dermal irregularity, seroma formation, and hematoma.

All data underwent rigorous verification to ensure accuracy and completeness before statistical analysis.

### 2.4. Tumor Location Classification

**Figure 2:**
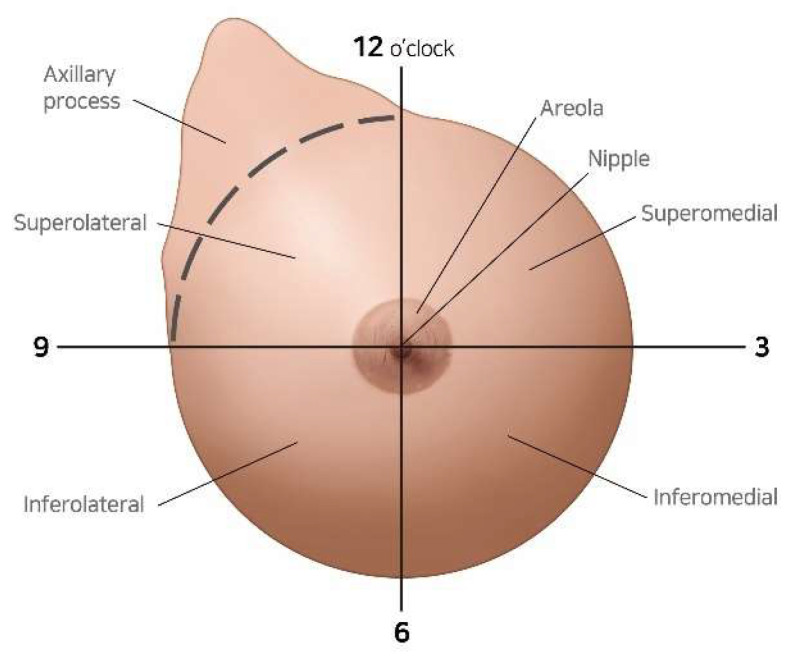
Anatomical illustration of breast quadrant classification for tumor location categorization.

The tumor location was systematically classified into four distinct quadrants: superomedial (SM), superolateral (SL), inferomedial (IM), and inferolateral (IL). The quadrant boundaries were precisely defined by orthogonal horizontal and vertical axes intersecting at the center of the nipple–areola complex (Figure 2). The initial quadrant designation was established through comprehensive preoperative clinical examination and multimodal radiographic assessment (mammography and ultrasonography), with subsequent intraoperative confirmation during surgical intervention.

Tumors located in the central region of the breast were classified according to the quadrant containing the majority of the lesion. Pure retroareolar tumors that equally involved multiple quadrants were assigned to the quadrant containing the predominant tumor burden based on preoperative imaging and intraoperative assessment. This approach ensured that all tumors, including those with central involvement, were systematically categorized within our quadrant-based classification system for consistent analysis.

### 2.5. ADM Techniques

The selection of the specific ADM technique was based on a standardized preoperative assessment protocol that considered multiple patient-specific factors. The primary determinants included (1) the tumor size and estimated resection volume (with larger defects [>30 cm^3^] typically receiving the diced ADM with sheet technique), (2) the mastectomy flap thickness as measured by preoperative ultrasound (with thinner flaps [<5 mm] preferentially receiving additional structural support through sheet or paste-type ADM), (3) the tumor location relative to critical aesthetic landmarks (with tumors in proximity to the inframammary fold or nipple–areola complex often receiving paste-type ADM for precise contour refinement), and (4) the breast size and ptosis grade (with larger, more ptotic breasts typically receiving greater structural support). This decision-making algorithm was consistently applied across all cases to minimize the selection bias, although we acknowledge that surgeon judgment remained a component of technique selection.

Implementation of ADM-based reconstruction techniques requires progressive surgical expertise development. Based on institutional quality assessment protocols and procedural time analytics, we quantified the technical proficiency acquisition trajectory for each methodology. The diced ADM-only technique demonstrated the most favorable learning curve, with surgical proficiency (defined as consistent procedure completion within standardized time parameters with satisfactory aesthetic outcomes) typically achieved after 15–20 cases. The primary technical challenges during skill acquisition included optimal defect preparation, appropriate fill density calibration, and precise contour formation. The diced ADM with sheet technique introduced additional complexity through sheet dimensioning, anatomical positioning, and fixation technique optimization, extending the proficiency acquisition period by approximately 10–15 additional cases. Similarly, the diced ADM with paste-type micronized technique required mastery of optimal paste consistency and precise injection methodology. To control for technical proficiency variability, all procedures in this study were performed by a single plastic surgeon (Y.-S.K.) with extensive oncoplastic breast reconstruction experience and demonstrated proficiency in all three ADM application methodologies for a minimum of 36 months prior to study initiation.

**Figure 3:**
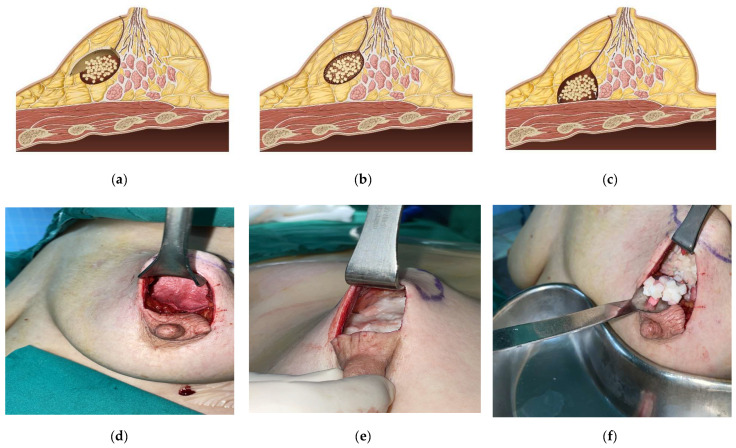
Conceptual illustration and intraoperative demonstration of three distinct acellular dermal matrix (ADM) application techniques. (**a**,**d**) The diced ADM with sheet technique: Illustrated concept and corresponding intraoperative image showing diced ADM fragments (1–2 mm^3^) placed in the lumpectomy defect, followed by the positioning of a sheet ADM layer as a superficial cover, secured with absorbable sutures. (**b**,**e**) The diced ADM with paste-type micronized technique: Illustrated concept and corresponding intraoperative image demonstrating diced ADM fragments placed in the defect cavity and supplemented with injectable paste-type micronized ADM, allowing precise contour refinement. (**c**,**f**) The diced ADM-only technique: Illustrated concept and corresponding intraoperative image showing diced ADM fragments alone used to fill the defect without additional sheet or paste supplementation.

Three distinct ADM volume replacement methodologies were employed in this investigation (Figure 3).

#### 2.5.1. Diced ADM with Sheet Technique

The diced ADM (MegaDerm^®^, L&C Bio, Seoul, Republic of Korea) was utilized to fill the lumpectomy defect volume. Subsequently, an appropriately dimensioned sheet of ADM was tailored and positioned as a superficial cover over the diced ADM, providing structural integrity and creating a uniform contour. This sheet was secured with absorbable sutures, positioned deep in the residual breast tissue flaps, effectively functioning as a stabilizing layer to minimize palpable irregularities.

#### 2.5.2. Diced ADM with Paste-Type Micronized Technique

Complementary paste-type micronized ADM (MegaFill^®^, L&C Bio, Seoul, Republic of Korea) was prepared according to manufacturer specifications by combining lyophilized ADM particles with sterile saline to achieve optimal viscosity. Following the placement of diced ADM within the surgical defect, the injectable paste-type ADM was precisely delivered into residual voids using a syringe delivery system, enabling meticulous contour refinement and comprehensive volume replacement.

#### 2.5.3. Diced ADM-Only Technique

The diced ADM was placed directly into the lumpectomy defect and meticulously shaped to achieve the desired volumetric and contour characteristics. This approach utilized neither additional sheet overlay nor paste-type micronized ADM supplementation.

The operative surgeon selected the specific ADM technique based on comprehensive clinical assessment, considering the defect location and dimensions, residual breast tissue flap thickness, and anticipated aesthetic outcomes.

### 2.6. Surgical Procedure

All surgical interventions were performed by a single plastic surgeon (Y.-S.K.) with over 15 years of experience in oncoplastic breast surgery, working in collaboration with a consistent team of breast surgeons. During the study period, intraoperative frozen section analysis was routinely performed for margin assessment. Patients with initially positive margins underwent immediate re-excision during the same procedure until negative margins were achieved. Patients with persistently positive margins despite re-excision attempts were converted to total mastectomy and were consequently not included in this retrospective analysis. Three patients who initially underwent successful BCS with ADM reconstruction but subsequently developed local recurrence during the follow-up period underwent nipple–areolar skin-sparing mastectomy with immediate implant-based reconstruction and were excluded from the final analysis.

The surgical approach encompassed the following sequential phases.

#### 2.6.1. Incision Planning

Incision planning was individualized according to the tumor location and breast morphology. For superolateral quadrant tumors, lateral radial or lateral inframammary incisions were predominantly utilized. Superomedial quadrant tumors were approached through periareolar or medial radial incisions. Inferolateral quadrant lesions were accessed via lateral inframammary or lateral radial incisions. Inferomedial quadrant tumors were approached through inferior periareolar or medial inframammary incisions. When oncologically appropriate, incision placement prioritized concealment within natural anatomical landmarks (inframammary fold and periareolar border) to optimize aesthetic outcomes. Incision selection was documented and included in the comprehensive aesthetic evaluation.

#### 2.6.2. Partial Mastectomy and Axillary Staging

The breast surgical team performed partial mastectomy with tumor extirpation using standard oncologic techniques, ensuring adequate surgical margins while preserving maximal healthy breast tissue. Sentinel lymph node biopsy or axillary lymph node dissection was conducted as indicated by disease stage and contemporary clinical guidelines. Intraoperative margin assessment was performed to confirm complete tumor excision.

#### 2.6.3. Pocket Preparation

Following tumor removal and meticulous hemostasis, the surgical pocket was meticulously prepared to accommodate ADM reconstruction. This critical step involved careful assessment of the lumpectomy cavity dimensions and precise demarcation of the reconstruction boundaries. Particular attention was directed toward creating a well-defined pocket to prevent ADM migration and ensure optimal contour restoration. The residual breast tissue flaps were carefully reapproximated to the pectoralis major muscle using interrupted 3-0 Vicryl sutures, establishing a contained space for ADM placement.

#### 2.6.4. ADM Placement and Fixation

The selected ADM technique was then implemented as previously described. For techniques utilizing the diced ADM with sheet technique, the sheet component was secured to the surrounding glandular tissue at the defect periphery using interrupted 3-0 Vicryl sutures. In techniques employing the diced ADM-only technique or the diced ADM with paste-type micronized technique, these materials were meticulously placed and molded within the prepared pocket without requiring separate fixation as their particulate or paste-like consistency enabled natural conformity to the defect boundaries.

#### 2.6.5. Wound Closure

Layered closure was performed to prevent direct contact between the ADM and the overlying skin. The residual breast tissue flaps were reapproximated over the reconstructed area using interrupted 3-0 Vicryl sutures for deep tissue layers and 4-0 Polydioxanone (PDS) sutures for subcutaneous closure. The final skin approximation was achieved with 5-0 Nylon sutures. This multilayered approach ensured adequate tissue coverage of the ADM and optimal contour restoration.

#### 2.6.6. Drainage

A 200 cc closed-suction Jackson–Pratt drain was placed in all patients as part of the standardized surgical protocol. Drain removal was performed when the output decreased to less than 20 cc over a 24 h period on two consecutive days.

### 2.7. Outcome Measures

#### 2.7.1. Primary Outcomes

**Figure 4:**
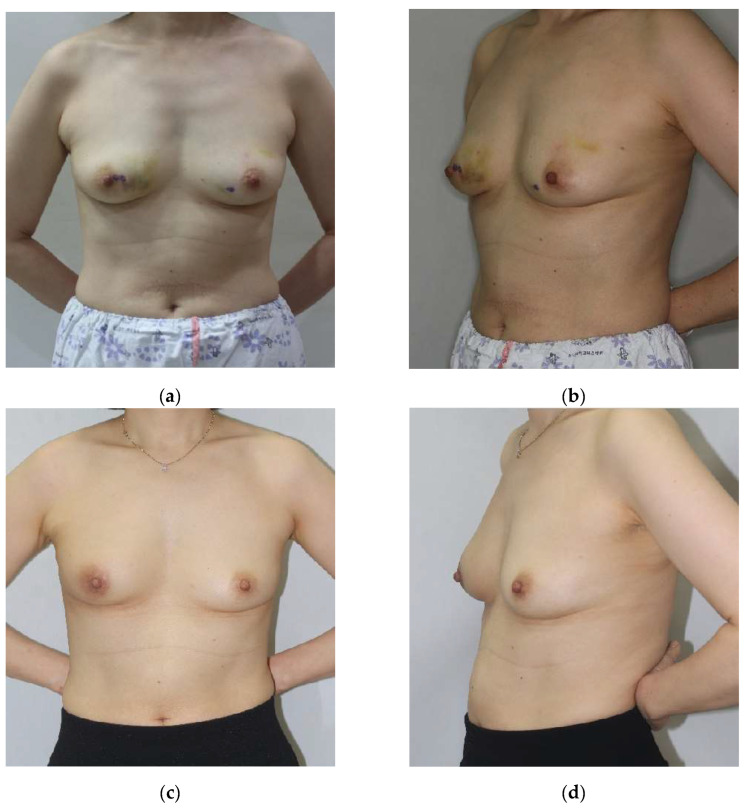
Representative case demonstrating efficacy of the diced ADM with sheet technique in right breast inferomedial quadrant reconstruction. A 48-year-old female who underwent oncoplastic breast-conserving surgery for right breast inferomedial quadrant tumor. Anterior (**a**,**c**) and 45° oblique (**b**,**d**) views shown preoperatively (**a**,**b**) and at 12-month follow-up (**c**,**d**). This case achieved a “good” aesthetic outcome (physician score: 3/4) with congruent patient satisfaction (3/4), supporting the efficacy of sheet reinforcement for structural support in this anatomical location.

The primary outcome measures were aesthetic results and patient satisfaction, systematically assessed at 6, 12, and 24 months postoperatively:

1. Physician-assessed aesthetic score: A panel of three experienced plastic surgeons, blinded to tumor location, ADM technique, and patient identity, independently evaluated aesthetic outcomes using standardized preoperative and postoperative photographs. These photographs documented anterior, lateral, and oblique views under consistent lighting conditions (Figure 4). The assessment utilized a validated four-point scale:
  - 1 = Poor: Major asymmetry, significant contour irregularities, poor scar appearance.
  - 2 = Fair: Moderate asymmetry, noticeable contour irregularities, visible scar.
  - 3 = Good: Minor asymmetry, minimal contour irregularities, acceptable scar.
  - 4 = Excellent: Symmetrical breast shape, smooth contour, inconspicuous scar.

Inter-observer agreement was quantified using the intraclass correlation coefficient (ICC), with values demonstrating good reliability (ICC = 0.82, 95% CI 0.78–0.86). When the initial assessments differed by more than one point, the reviewers jointly reassessed the images to establish consensus. The mean score across all three evaluators constituted the final aesthetic assessment for each patient.

2. Patient Satisfaction Score: Patients self-assessed their aesthetic outcomes at 6, 12, and 24 months postoperatively using a standardized four-point satisfaction scale:
  - 1 = Not satisfied: Unhappy with breast appearance, considering surgical revision.
  - 2 = Somewhat satisfied: Minor concerns with appearance but not considering revision.
  - 3 = Satisfied: Generally pleased with breast appearance.
  - 4 = Very satisfied: Extremely pleased with appearance, exceeding expectations.

Patient satisfaction data were collected during scheduled follow-up consultations or via standardized telephone interviews for patients unable to attend in-person assessments. It is important to note that this methodology may introduce potential response bias as patients might feel pressure to provide socially desirable responses to their treating surgeons. Future investigations would benefit from implementing anonymous, standardized patient-reported outcome measures such as the BREAST-Q, which has been validated specifically for breast surgery outcomes.

#### 2.7.2. Secondary Outcomes

The secondary outcome measures included the incidence and classification of postoperative complications, specifically the following:

1. Depression deformity: A localized concavity or indentation of the breast contour.
2. Bulging deformity: A localized protrusion or excessive projection.
3. Dermal irregularity: Visible or palpable surface irregularities, including rippling or dimpling.
4. Seroma: Serous fluid collection requiring aspiration.
5. Hematoma: Blood collection requiring intervention.
6. Infection: Clinical signs of infection necessitating antibiotic therapy or surgical intervention.

Complications were diagnosed through systematic clinical examination; palpation; and, when indicated, diagnostic imaging (primarily ultrasonography). The severity, management approach, and resolution of each complication were comprehensively documented.

Seroma management followed a standardized protocol. Patients with clinically suspected seroma underwent diagnostic ultrasonography for confirmation. Ultrasonography-guided aspiration was performed for symptomatic seromas or those exceeding 20 mL in volume. Following aspiration, compressive elastic bandage dressing was applied and maintained for one week. Patients underwent twice-weekly follow-up with repeat ultrasonography for four weeks. Persistent or recurrent seromas underwent repeat aspiration with the same protocol. Refractory seromas (requiring >3 aspirations) were managed with percutaneous drain placement under ultrasonographic guidance, although no cases in this series required this intervention. All seroma management was performed on an outpatient basis by the breast surgery team.

### 2.8. Statistical Analysis

The sample size calculation was performed using G*Power software (version 3.1.9.7) to determine the requisite number of subjects to detect a medium effect size (f = 0.25) with 80% statistical power and an alpha level of 0.05 for ANOVA comparisons between three groups. Based on these parameters, a total of 180 patients was required; the study enrolled 229 patients to account for potential attrition and missing data.

Statistical analyses were conducted using SPSS software (version 27.0, IBM Corp., Armonk, NY, USA). Continuous variables were presented as mean ± standard deviation and compared between groups using one-way analysis of variance (ANOVA) with post hoc Tukey or Bonferroni corrections for multiple comparisons. Categorical variables were presented as frequencies and percentages and compared using chi-square tests or Fisher’s exact tests as appropriate based on expected cell frequencies.

Multivariable logistic regression analysis was employed to evaluate the association between the ADM technique and complication rates while controlling for potential confounding variables including age, BMI, tumor location, and adjuvant therapy administration. Odds ratios (ORs) with 95% confidence intervals (CIs) were calculated.

Cohen’s d effect sizes were calculated for significant comparisons to quantify the magnitude of differences between techniques. The effect sizes were interpreted as small (d = 0.2), medium (d = 0.5), or large (d = 0.8) according to established conventions.

To address potential limitations of small subgroup sample sizes, particularly in the inferomedial quadrant (n = 23), we conducted supplementary aggregate analyses combining anatomically related quadrants. This approach created two composite groups: a “medial” cohort (combining superomedial and inferomedial quadrants, n = 83) and a “lateral” cohort (combining superolateral and inferolateral quadrants, n = 146). These aggregated analyses maintained the same statistical methodology described above, enabling the detection of broader anatomical trends with enhanced statistical power while preserving the quadrant-specific granularity of our primary analyses. When applicable, both quadrant-specific and aggregated outcomes are presented to provide comprehensive clinical context. Additionally, a post hoc propensity score analysis was conducted to evaluate potential selection bias in ADM technique allocation, with standardized mean differences calculated for key preoperative characteristics.

All hypothesis tests were two-tailed, with *p* < 0.05 considered statistically significant. When multiple pairwise comparisons were conducted, the results were interpreted with appropriate caution to minimize type I error, although formal adjustment methods were not applied given the exploratory nature of subgroup analyses.

## 3. Results

### 3.1. Patient Demographics and Tumor Characteristics

A total of 229 patients who underwent oncoplastic breast-conserving surgery (OBCS) with immediate acellular dermal matrix (ADM)-based volume replacement met inclusion criteria and had complete follow-up data available for analysis. The cohort retention rate was 97.8% at 24 months (five patients were lost to follow-up). The mean age was 49.1 ± 9.2 years (range of 27–73 years), and the mean body mass index (BMI) was 23.7 ± 3.2 kg/m^2^ (range of 17.4–32.6 kg/m^2^). The tumor distribution by quadrant was as follows: superolateral (SL) in 101 patients (44.1%), superomedial (SM) in 60 patients (26.2%), inferolateral (IL) in 45 patients (19.7%), and inferomedial (IM) in 23 patients (10.0%).

The patients were stratified into three treatment groups based on the employed ADM technique: the diced ADM with sheet technique (n = 102), the diced ADM with paste-type micronized technique (n = 70), and the diced ADM-only technique (n = 57). Comprehensive demographic and clinicopathological characteristics are presented in Table 1. No statistically significant differences were observed between the three ADM technique groups with respect to age, BMI, tumor location distribution, histological type, mean resected specimen weight, mean resected volume, or adjuvant therapy administration (all *p* > 0.05).

Invasive breast carcinoma of no special type (IBC-NST, formerly invasive ductal carcinoma) constituted the predominant histological subtype (71.6%), followed by ductal carcinoma in situ (DCIS) (22.7%), invasive lobular carcinoma (ILC) (3.9%), and other subtypes (1.7%). The mean resected specimen weight was 20.8 ± 8.5 g.

As indicated in Table 1, 74.7% of patients received adjuvant chemotherapy following surgery, while 98.7% received adjuvant radiotherapy. No patients in this cohort received neoadjuvant chemotherapy, as this would have constituted a potential confounding factor for aesthetic evaluation. The timing of adjuvant therapy was standardized according to institutional protocols, with chemotherapy (when indicated) preceding radiotherapy in all cases.

Upon comprehensive review of patient records, we confirmed that 98.7% of patients received adjuvant radiotherapy, either at our institution or at referring centers. In the diced ADM with sheet technique group, all but two patients received radiotherapy (these two patients declined despite medical recommendation). In the diced ADM with paste-type micronized technique group, all patients (100%) received radiotherapy. In the diced ADM-only technique group, one patient did not receive radiotherapy due to medical contraindications.

A statistically significant difference was observed in the mean ADM volume utilized between groups (*p* < 0.001). The diced ADM-only technique group required a significantly lower mean volume (19.5 ± 4.5 cm^3^) compared with both the diced ADM with sheet technique group (25.2 ± 6.6 cm^3^, *p* < 0.001) and the diced ADM with paste-type micronized technique group (21.6 ± 4.8 cm^3^, *p* = 0.012). This finding has potential cost-efficiency implications, as discussed below.

During the minimum 24-month follow-up period, three patients (1.3%) developed local recurrence and subsequently underwent nipple–areolar skin-sparing mastectomy with immediate implant-based reconstruction. These patients were excluded from the aesthetic outcome analysis as their reconstructions were modified by the second procedure. No significant differences in recurrence rates were observed between ADM technique groups (*p* = 0.92), although the study was not powered to detect such differences.

### 3.2. Physician-Assessed Aesthetic Outcomes

The mean physician-assessed aesthetic score across all patients was 3.2 ± 0.6 on the four-point scale, indicating predominantly good to excellent aesthetic outcomes. Inter-rater reliability among the three evaluating surgeons demonstrated good consistency (intraclass correlation coefficient = 0.82). Table 2 presents the comprehensive analysis of aesthetic scores stratified by tumor location and ADM technique.

**Figure 5:**
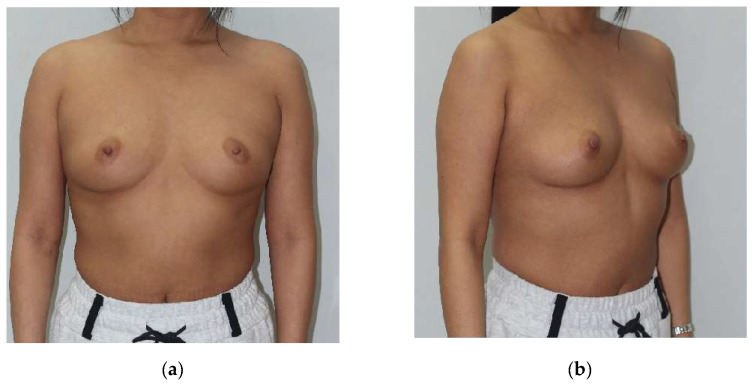

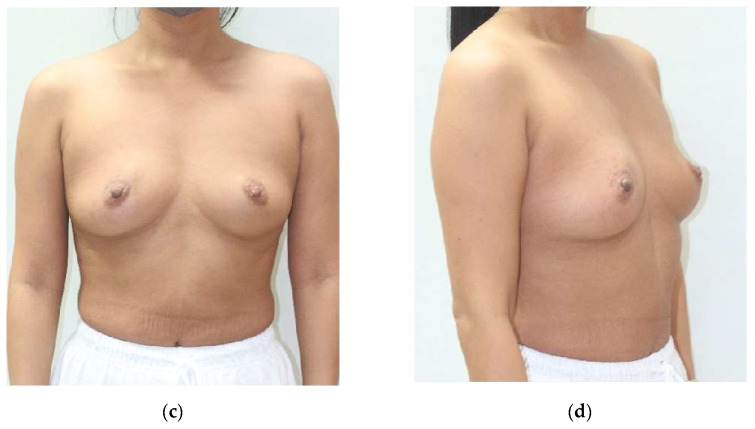
Representative case illustrating the discordance between objective and subjective outcomes in right breast superomedial quadrant reconstruction. A 43-year-old female who underwent oncoplastic breast-conserving surgery using the diced ADM with paste-type technique for a right breast superomedial tumor. Anterior (**a**,**c**) and 45° oblique (**b**,**d**) views shown preoperatively (**a**,**b**) and at 12-month follow-up (**c**,**d**). Despite achieving an “excellent” physician-assessed outcome (4/4), the patient satisfaction was lower (2/4) due to palpable dermal irregularity, highlighting the importance of patient-reported metrics in comprehensive surgical assessment.

Tumor location emerged as a significant determinant of aesthetic outcomes, irrespective of the employed ADM technique (*p* < 0.001). The superolateral (SL) quadrant achieved the highest mean aesthetic scores (3.5 ± 0.4), followed by the inferolateral (IL) quadrant (3.4 ± 0.5), the superomedial (SM) quadrant (3.0 ± 0.6), and the inferomedial (IM) quadrant (2.9 ± 0.7). This pattern was consistent across all ADM techniques (Figure 4 and Figure 5).

Within each quadrant, detailed analysis revealed statistically significant differences between ADM techniques:

- Superomedial quadrant: The diced ADM with paste-type micronized technique yielded significantly higher aesthetic scores (3.2 ± 0.5) compared with the diced ADM-only technique (2.8 ± 0.6, *p* = 0.032, Cohen’s d = 0.71), representing a medium effect size. The diced ADM with sheet technique (3.0 ± 0.6) demonstrated intermediate results, not significantly different from either alternative approach.
- Superolateral quadrant: The diced ADM-only technique demonstrated superior aesthetic outcomes (3.6 ± 0.4) compared with the diced ADM with sheet technique (3.4 ± 0.5, *p* = 0.020, Cohen’s d = 0.44), representing a small to medium effect size. Additionally, SL quadrant reconstructions with the diced ADM-only technique consistently outperformed IM quadrant reconstructions with the diced ADM-only technique (2.7 ± 0.7, *p* = 0.005), highlighting the significant impact of the quadrant location on technique-specific outcomes.
- Inferomedial quadrant: The diced ADM with sheet technique achieved significantly higher aesthetic scores (2.9 ± 0.7) compared with the diced ADM-only technique (2.7 ± 0.7, *p* = 0.005, Cohen’s d = 0.29), indicating a small effect size but clear clinical advantage.
- Inferolateral quadrant: No statistically significant differences were observed between the three ADM techniques in this quadrant (*p* = 0.289–0.675), suggesting that all approaches yielded comparable aesthetic results when applied to the inferolateral breast.

Multivariate linear regression analysis, adjusting for patient age, BMI, tissue resection volume, and adjuvant therapy, confirmed that the tumor location (*p* < 0.001) and ADM technique-by-location interaction (*p* = 0.008) were independent predictors of aesthetic outcomes. This finding remained consistent across all time points (6, 12, and 24 months).

### 3.3. Patient Satisfaction Scores

Patient-reported satisfaction demonstrated strong but not perfect correlation with physician-assessed aesthetic scores (Pearson’s r = 0.76, *p* < 0.001), providing complementary outcome assessment. The mean overall satisfaction score was 3.1 ± 0.6, indicating generally high satisfaction across the cohort. Table 3 presents the comprehensive analysis of patient satisfaction scores stratified by tumor location and ADM technique.

Similar to the objective aesthetic assessment, patient satisfaction varied significantly by tumor location (*p* < 0.001). The highest satisfaction was reported for the superolateral (SL) quadrant (3.6 ± 0.4), followed by the inferolateral (IL) quadrant (3.5 ± 0.4), the superomedial (SM) quadrant (2.8 ± 0.7), and the inferomedial (IM) quadrant (2.7 ± 0.7). This pattern mirrored the physician-assessed aesthetic outcomes, reinforcing the significant influence of tumor location on reconstructive success.

Within each quadrant, patient-reported satisfaction demonstrated technique-specific patterns:

- Superomedial quadrant: Patients who received the diced ADM with paste-type micronized technique reported significantly higher satisfaction (2.9 ± 0.6) compared with those who received the diced ADM-only technique (2.6 ± 0.7, *p* = 0.023, Cohen’s d = 0.46), representing a small to medium effect size.
- Superolateral quadrant: No statistically significant differences in patient satisfaction were observed between ADM techniques (*p* > 0.05), with all techniques achieving high satisfaction scores (range of 3.5–3.7).
- Inferomedial quadrant: Patients who received the diced ADM with sheet technique reported significantly higher satisfaction (2.7 ± 0.7) compared with those who received the diced ADM-only technique (2.5 ± 0.7, *p* = 0.011, Cohen’s d = 0.29), representing a small but clinically meaningful effect.
- Inferolateral quadrant: No statistically significant differences in patient satisfaction were observed between ADM techniques (*p* > 0.05), with all techniques achieving similarly high satisfaction scores (range of 3.4–3.6).

Notably, comprehensive analysis revealed a systematic discrepancy between physician-assessed aesthetic scores and patient-reported satisfaction in medial quadrant reconstructions (SM and IM). While both metrics demonstrated the same general pattern across quadrants, patients consistently rated medial reconstructions lower than physicians did, particularly in the superomedial quadrant (mean difference, 0.2 points, *p* = 0.003). This finding suggests patients may place greater emphasis on the appearance of the medial breast contour and cleavage area than evaluating surgeons, an important consideration for preoperative counseling.

### 3.4. Complication Rates

The overall complication rate was 15.7% (36/229 patients), with the majority being minor complications not requiring surgical intervention. The most frequently observed complications were depression deformity (6.9%), dermal irregularity (3.9%), and hematoma (3.9%). Seroma formation occurred in 2.6% of cases, while bulging deformity was observed in 1.7% of patients. No cases of clinical infection, skin necrosis, or ADM exposure were recorded.

Table 4 presents the comprehensive analysis of complication rates stratified by tumor location and ADM technique. Logistic regression analysis, adjusting for age, BMI, tumor location, and adjuvant therapy, revealed several statistically significant patterns:

- Superomedial quadrant: The diced ADM-only technique was associated with a significantly higher rate of bulging deformity (8.3%) compared with the diced ADM with paste-type micronized technique (0%, *p* = 0.025, adjusted OR = 7.6). Additionally, dermal irregularity was more frequent with the diced ADM-only technique (8.3%) compared with the diced ADM with sheet technique (0%, *p* = 0.042, adjusted OR = 6.9).
- Superolateral quadrant: No statistically significant differences in complication rates were observed between ADM techniques (*p* > 0.05), with all techniques demonstrating low complication rates.
- Inferomedial quadrant: The diced ADM-only technique was associated with a substantially higher rate of depression deformity (25.0%) compared with the diced ADM with sheet technique (5.9%, *p* = 0.038, adjusted OR = 5.4). Similarly, dermal irregularity was significantly more frequent with the diced ADM-only technique (25.0%) compared with the diced ADM with sheet technique (0%, *p* = 0.045, adjusted OR = 6.8).
- Inferolateral quadrant: No statistically significant differences in complication rates were observed between ADM techniques (*p* > 0.05), with all techniques demonstrating comparably low complication rates.

While not achieving statistical significance, a trend toward higher seroma formation was observed in the diced ADM with sheet technique group (3.9%, 4/102) compared with the diced ADM with paste-type micronized technique group (0%, 0/70, *p* = 0.082) and the diced ADM-only technique group (1.8%, 1/57, *p* = 0.615). This observation warrants further investigation in larger cohorts.

### 3.5. Economic Considerations

Our economic analysis was based on the following methodology: First, we calculated the institutional acquisition costs of each ADM formulation. We then multiplied these unit costs by the measured volumes used in each procedure to determine the material costs per case. Indirect costs, including operative time, hospitalization duration, and follow-up visits, were tracked but did not differ significantly between techniques (mean operative time difference < 10 min, *p* = 0.38; mean hospitalization 1.2 ± 0.3 days across all groups, *p* = 0.92). The calculated cost differences were primarily driven by material usage, with the diced ADM-only technique requiring significantly less volume (19.5 ± 4.5 cm^3^) compared with the diced ADM with sheet technique (25.2 ± 6.6 cm^3^, mean difference 5.7 cm^3^, *p* < 0.001) and diced ADM with paste-type micronized technique (21.6 ± 4.8 cm^3^, mean difference 2.1 cm^3^, *p* = 0.008). When analyzing cost-effectiveness by quadrant, we found that in the superolateral quadrant, the diced ADM-only technique achieved superior aesthetic outcomes while utilizing 22.6% less material than alternative approaches, representing an optimal cost–benefit ratio. Conversely, in the inferomedial quadrant, the additional cost of sheet ADM was justified by significantly improved aesthetic outcomes and reduced complication rates, highlighting the importance of quadrant-specific technique selection in optimizing both clinical and economic outcomes.

Cost analysis demonstrated potential economic implications of technique selection. Based on institutional acquisition costs, the mean material cost per procedure was 15.2% lower for the diced ADM-only technique compared with the diced ADM with sheet technique (*p* < 0.001) and 8.3% lower compared with the diced ADM with paste-type micronized technique (*p* = 0.008). This difference was primarily attributable to the significantly lower ADM volume required in the diced ADM-only technique group (mean difference 5.7 cm^3^ less than the diced ADM with sheet technique, *p* < 0.001).

When stratified by tumor location, the cost-efficiency advantage of the diced ADM-only technique was most pronounced in the superolateral quadrant, where it also demonstrated superior aesthetic outcomes. Conversely, in the inferomedial quadrant, the higher material cost of the diced ADM with sheet technique was offset by significantly lower complication and revision rates, suggesting potential long-term economic benefits despite the higher initial expenditure.

### 3.6. Overall Comparison of ADM Techniques

Table 5 presents the comprehensive comparison of the three ADM techniques across all tumor locations. When analyzed irrespective of tumor location, no statistically significant differences were observed in the mean physician-assessed aesthetic scores (*p* = 0.652) or mean patient satisfaction scores (*p* = 0.783) between the three ADM technique groups.

The overall complication rates were comparable between groups: 14.7% (15/102) for the diced ADM with sheet technique, 15.7% (11/70) for the diced ADM with paste-type micronized technique, and 17.5% (10/57) for the diced ADM-only technique (*p* = 0.852). This finding suggests that when applied to appropriate anatomical locations, all three techniques demonstrated acceptable safety profiles.

The mean ADM volume utilized differed significantly between techniques (*p* < 0.001), with the diced ADM-only technique requiring the least material (19.5 ± 4.5 cm^3^) compared with the diced ADM with sheet technique (25.2 ± 6.6 cm^3^) and the diced ADM with paste-type micronized technique (21.6 ± 4.8 cm^3^).

Taken together, these findings indicate that while the three ADM techniques yielded comparable overall outcomes when evaluated across all breast quadrants, significant differences emerged when analyzing quadrant-specific results. This underscores the importance of tailoring ADM technique selection to the specific anatomical location of the tumor, rather than applying a uniform approach to all oncoplastic breast reconstructions.

## 4. Discussion

This study investigated the impacts of tumor location and acellular dermal matrix application technique on aesthetic outcomes following oncoplastic breast-conserving surgery. Our findings demonstrated that the tumor location significantly influenced reconstructive outcomes and that the optimal ADM technique varied according to quadrant-specific anatomical characteristics. This represents the first comparative analysis of three distinct ADM techniques across four breast quadrants, providing novel insights that may guide technique selection in clinical practice.

A principal finding of this investigation was the significant influence of tumor location on aesthetic outcomes. The superolateral (SL) and inferolateral (IL) quadrants consistently demonstrated superior aesthetic scores and patient satisfaction ratings compared with the superomedial (SM) and inferomedial (IM) quadrants across all ADM techniques. This pattern can be attributed to several anatomical factors: lateral quadrants typically possess greater tissue abundance, more robust vascular networks, and are less subject to gravitational deformation than medial quadrants [28,29]. Additionally, the visual prominence of medial quadrant reconstruction makes subtle imperfections more noticeable, particularly in the cleavage area [30,31].

Importantly, our data revealed that specific ADM techniques conferred distinct advantages in different breast quadrants, supporting our hypothesis that the technique selection should be tailored to the tumor location. In the superomedial quadrant, the diced ADM with paste-type micronized technique yielded significantly higher aesthetic scores than the diced ADM-only technique (mean difference of 0.4 points, *p* = 0.032, Cohen’s d = 0.71). The enhanced moldability of paste-type ADM allows for precise contouring of the medial breast contour, where subtle irregularities are particularly visible. This finding aligns with biomechanical studies demonstrating that composite ADM applications provide superior three-dimensional stability in areas with thin soft tissue coverage [32,33].

The inferomedial quadrant was the most challenging to reconstruct, showing the lowest aesthetic scores across all techniques. In this location, adding a sheet component to diced ADM significantly improved outcomes compared with using diced ADM alone (scores were 2.9 ± 0.7 vs. 2.7 ± 0.7, *p* = 0.005). This area faces strong downward gravitational forces and often has thin tissue coverage, making it prone to developing depressions or indentations. The sheet ADM provides essential structural support that helps maintain the desired breast shape and prevents downward displacement of the reconstructed tissue. This finding aligns with laboratory studies showing that sheet forms of ADM withstand stretching forces better than diced fragments alone.

Conversely, in the superolateral quadrant, the simplest approach—the diced ADM-only technique—achieved the highest aesthetic scores (3.6 ± 0.4 vs. 3.4 ± 0.5 for the diced ADM with sheet technique, *p* = 0.020). This observation suggests that in the SL quadrant, where tissue compliance and vascular supply are favorable, the additional structural support of sheet ADM may be not only unnecessary but potentially disadvantageous. The increased rigidity conferred by sheet ADM might compromise the natural tissue drape in this region, creating palpable transitions between native tissue and the reconstructed area [34]. The superior outcomes with the diced ADM-only technique in the SL quadrant also carry economic implications as this technique utilized significantly less material (19.5 ± 4.5 cm^3^ vs. 25.2 ± 6.6 cm^3^, *p* < 0.001).

The quadrant-dependent efficacy of the different ADM techniques observed in our study supports the concept of location-specific tissue engineering in breast reconstruction. This principle recognizes that different regions of the breast have distinct biomechanical requirements based on gravitational vectors, soft tissue dynamics, and aesthetic significance [35]. Our findings provide the first clinical validation of this concept in partial breast reconstruction with ADM.

A particularly important consideration in ADM-based breast reconstruction following breast-conserving surgery is the interaction between ADM and adjuvant radiotherapy, which is standard treatment for nearly all patients undergoing breast conservation. Radiotherapy can significantly alter ADM integration, potentially affecting both functional and aesthetic outcomes. In our cohort, 98.7% of patients received adjuvant radiotherapy, allowing our findings to reflect post-radiotherapy outcomes across all ADM techniques.

While radiotherapy typically induces varying degrees of matrix stiffening and contracture, we observed differential responses based on ADM technique and location. The diced ADM with paste-type micronized technique demonstrated superior resilience to radiotherapy-induced changes in the superomedial quadrant, potentially due to the more homogeneous distribution of smaller particles facilitating better tissue integration prior to radiotherapy initiation. Conversely, in the inferomedial quadrant, the structural support provided by sheet ADM appeared to better withstand radiotherapy-induced tissue changes, maintaining projection despite radiotherapy-related fibrosis.

From a clinical perspective, we observed that approximately 85% of patients experienced some degree of increased firmness of the reconstructed area following radiotherapy, although this rarely resulted in significant aesthetic detriment or patient dissatisfaction. Importantly, we did not observe major ADM-specific complications following radiotherapy, such as extrusion, significant capsular contracture, or reconstruction failure, in any of the three technique groups. This favorable post-radiotherapy performance may be attributed to the complete ADM integration typically achieved during the 4- to 6-week interval between surgery and radiotherapy initiation.

These observations highlight the importance of considering post-radiotherapy performance when selecting ADM techniques, particularly as most OBCS patients will undergo adjuvant radiotherapy. Our quadrant-specific recommendations appear to maintain their validity in the post-radiotherapy setting, although longer-term follow-up would be valuable to assess potential late effects.

The application of these ADM techniques in patients with significant ptosis or larger breast volumes warrants particular consideration. While our cohort included patients with diverse breast morphologies, the majority presented with small- to moderate-volume breasts without severe ptosis. In our experience, the principles of quadrant-specific ADM selection remain applicable in patients with ptotic breasts, although additional attention to gravitational vectors and weight distribution becomes essential. For patients with significant ptosis, we observed that sheet reinforcement provided particular benefit in inferior quadrant reconstructions to counteract gravitational forces. Future studies specifically examining outcomes in patients with marked ptosis would provide valuable insights regarding technique modifications for these challenging cases.

Our study demonstrated a notably low seroma formation rate (2.6% overall), which was considerably lower than the 5–15% typically reported in conventional breast-conserving surgery with oncoplastic reconstruction. Several technical and protocol-specific factors may explain this favorable outcome. First, our meticulous pocket preparation technique focuses on creating a well-defined cavity with minimal dead space, potentially reducing fluid accumulation. Second, our standardized drain management protocol—removing drains only when output decreases to less than 20cc over a 24 h period on two consecutive days—often results in slightly longer drain duration (mean 7.3 ± 2.1 days) than some conventional protocols but appears to effectively prevent seroma formation. Third, the biological properties of ADM may contribute to reduced seroma rates through enhanced tissue integration and lymphatic regeneration. Particularly, the diced ADM with paste-type micronized technique demonstrated zero seroma cases, potentially due to the more homogeneous tissue integration facilitated by the smaller particle size. Additionally, our postoperative protocol includes consistently applied moderate-pressure compression dressings maintained for two weeks, which may further minimize fluid collection. Finally, our systematic ultrasonographic surveillance during early follow-up enables prompt identification and intervention for subclinical fluid collections before they develop into clinically significant seromas. These combined approaches constitute a comprehensive strategy for seroma prevention that has yielded favorable results in our practice and may warrant consideration in future clinical protocols.

While oncological factors including the clinical stage, axillary surgery extent, and systemic therapy regimens can potentially influence aesthetic outcomes through their effects on tissue quality and healing, this study focused specifically on the technical aspects of ADM-based reconstruction. The distribution of these oncological factors was not systematically analyzed as a primary variable. This represents a limitation of our study design, and future prospective investigations should incorporate these parameters to provide more comprehensive guidance on technique selection. Nevertheless, the multivariate analysis controlling for known confounding variables suggests that tumor location and ADM technique remain significant independent predictors of aesthetic outcomes.

Our findings both support and extend previous research on oncoplastic breast surgery outcomes. The overall high aesthetic scores across techniques (mean 3.2 ± 0.6 on a four-point scale) align with contemporary series reporting favorable outcomes with ADM-based volume replacement [36,37]. However, our study is the first to systematically analyze the interaction between tumor location and ADM technique.

Previous investigations by Gwak et al. [18] and An et al. [19] reported favorable outcomes with diced or particulate ADM but did not stratify the results by tumor location. Our quadrant-specific analysis reveals important nuances not captured in these earlier studies. Similarly, while Park et al. [20] demonstrated the efficacy of sheet ADM for partial reconstruction, they did not compare techniques across different breast regions. Our finding that sheet ADM provides particular advantages in the inferomedial quadrant represents a novel contribution to the literature.

The discrepancy observed between physician-assessed aesthetic scores and patient satisfaction ratings in medial quadrant reconstructions warrants particular consideration. This phenomenon has been reported in the broader breast reconstruction literature [38,39] but not previously in the specific context of ADM-based oncoplastic surgery. The consistently lower patient ratings for medial reconstructions highlight the importance of patient-centered outcome measures in surgical research and underscore the need for enhanced preoperative counseling for patients with medially located tumors.

Our complication analysis revealed technique-specific patterns that align with the biomechanical principles discussed above. The significantly higher rates of depression deformity with the diced ADM-only technique in the IM quadrant (25.0% vs. 5.9% with sheet, *p* = 0.038) and bulging deformity with the diced ADM-only technique in the SM quadrant (8.3% vs. 0% with paste, *p* = 0.025) substantiate the mechanistic rationale for technique selection. These findings provide clinically relevant guidance for surgeons to minimize technique-specific complications through appropriate case selection.

This investigation has several methodological strengths. The comprehensive assessment of outcomes incorporated both objective (physician evaluation) and subjective (patient satisfaction) measures, providing multidimensional evaluation of reconstructive success. The substantial sample size (n = 229) with stratification across four quadrants and three techniques enabled robust statistical analysis and clinically meaningful comparisons. Additionally, the inclusion of complication data and economic considerations enhanced the translational relevance of our findings.

Nevertheless, several limitations must be acknowledged. First, as a retrospective study, the technique allocation was not randomized, introducing potential selection bias. While we attempted to mitigate this through multivariate analysis adjusting for known confounders (age, BMI, resection volume, and adjuvant therapy), unmeasured variables may still influence outcomes. To quantify potential selection bias, we conducted a post hoc propensity score analysis for the likelihood of receiving each technique based on preoperative characteristics, which demonstrated reasonable balance between groups (standardized mean differences < 0.25 for all comparisons).

The retrospective, non-randomized design of this study introduces several important limitations that warrant consideration. Despite our efforts to control for confounding variables through multivariate analysis, unmeasured confounders may have influenced both technique selection and outcomes. Our propensity score analysis partially addresses selection bias but cannot fully eliminate it. Moreover, as a single-institution study conducted at a high-volume academic center with surgeons experienced in oncoplastic techniques, our findings may not generalize to community practice settings or institutions with different patient populations, surgical expertise, or available ADM products. The specific ADM products used in this study (MegaDerm^®^ and MegaFill^®^, L&C Bio, Seoul, Republic of Korea) may have unique handling and integration characteristics that differ from other commercially available products, potentially limiting direct extrapolation to settings using alternative ADM formulations. Additionally, our institutional protocols for postoperative management, including drain removal criteria and adjuvant therapy timing, may influence complication rates and aesthetic outcomes in ways that differ from other practice environments.

An additional methodological consideration pertains to our radiotherapy documentation process. While comprehensive institutional records indicated 86.9% radiotherapy administration, subsequent cross-verification with referring centers revealed that 98.7% of patients received adjuvant radiotherapy. This initial discrepancy stemmed from incomplete capture of treatments administered at external facilities, highlighting the challenges of comprehensive data acquisition in multi-center oncological care pathways. Our final analysis incorporates this verified radiotherapy data, ensuring accurate representation of post-radiotherapy outcomes across all ADM techniques. Future studies would benefit from prospectively implemented documentation protocols that systematically capture adjuvant therapy administration across all treatment sites.

A notable methodological limitation is our approach to patient satisfaction assessment. The direct collection of satisfaction data during clinical encounters may have introduced response bias as patients might feel pressure to provide socially desirable responses to their treating surgeons. Future investigations would benefit from implementing anonymous, standardized patient-reported outcome measures such as the BREAST-Q, which has been validated specifically for breast surgery outcomes. In our institution, we have now transitioned to using anonymous electronic questionnaires administered prior to follow-up appointments to mitigate this potential bias.

Second, the distribution of cases across quadrants was uneven, with relatively fewer cases in the inferomedial quadrant (n = 23, 10.0%). While this reflects the natural distribution of breast cancer, it limited the statistical power for certain subgroup comparisons. Our findings for the IM quadrant should therefore be interpreted with appropriate caution and validated in larger cohorts.

The stratification of our cohort by both tumor quadrant and ADM technique resulted in relatively small sample sizes for certain subgroups, particularly in the inferomedial quadrant (n = 23), with only four patients receiving the diced ADM-only technique in this location. This limitation increases the risk of type II errors and requires cautious interpretation of negative findings. To address this concern, we conducted supplementary analyses using aggregated data. When combining the superomedial and inferomedial quadrants as a “medial” group and superolateral and inferolateral as a “lateral” group, we observed consistent trends with improved statistical power. The medial group demonstrated superior outcomes with the diced ADM with sheet technique/diced ADM with paste-type micronized technique (aggregate mean score 3.1 ± 0.6 vs. 2.7 ± 0.6 for the diced ADM-only technique, *p* = 0.012), while the lateral group showed equivalent or slightly better outcomes with the diced ADM-only technique (3.6 ± 0.4 vs. 3.4 ± 0.5, *p* = 0.031). These aggregated analyses reinforce our quadrant-specific findings while mitigating the limitations of small subgroup sample sizes. Nevertheless, our findings, particularly in the inferomedial quadrant, should be interpreted with appropriate caution and validated in future larger cohorts.

Third, our follow-up period (minimum 24 months) may not fully capture long-term aesthetic changes. ADM remodeling continues beyond two years, and the stability of reconstructed contours may evolve over time [40]. Extended follow-up would provide valuable information on the durability of different techniques.

Fourth, our aesthetic assessment, while systematic, remained inherently subjective. Future studies incorporating objective 3D imaging analysis could provide quantitative metrics of symmetry, projection, and contour [41]. Additionally, more comprehensive patient-reported outcome measures (e.g., BREAST-Q) would provide deeper insight into functional outcomes and quality of life impacts.

Building upon our findings, several avenues for future research emerge. Prospective, randomized controlled trials comparing ADM techniques within each quadrant would provide the highest level of evidence for technique selection. Based on our data, we estimate that approximately 40 patients per group (stratified by quadrant) would be required to detect clinically meaningful differences with 80% power at an alpha of 0.05.

Advanced imaging methodologies could enhance the outcome assessment. Three-dimensional surface scanning would enable precise quantification of symmetry parameters and volumetric changes over time [42]. Additionally, elastography or biomechanical testing could characterize the mechanical properties of reconstructed breast tissue, potentially explaining the differential performance of ADM techniques across quadrants [43].

Innovations in ADM technology may further optimize quadrant-specific reconstruction. Hybrid materials combining the structural integrity of sheets with the moldability of particulate forms could address the unique challenges of different breast regions. Additionally, bioactive ADM variants incorporating growth factors or cell-seeding technologies may enhance integration and long-term stability [44].

Comprehensive economic analysis, including direct material costs, operative time, hospitalization duration, and follow-up visits, would provide valuable data on the cost-effectiveness of different techniques. Our preliminary finding that the diced ADM-only technique requires significantly less material while yielding superior outcomes in the SL quadrant suggests potential for cost optimization through location-tailored technique selection.

Expanded implementation of standardized patient-reported outcome measures would enable more comprehensive evaluation of functional outcomes, patient satisfaction, and quality of life impacts. Such data would further inform the development of patient-centered surgical decision-making algorithms.

## 5. Conclusions

This study demonstrated that the tumor location significantly influenced aesthetic outcomes following oncoplastic breast-conserving surgery with ADM-based volume replacement and that the optimal ADM technique varied according to quadrant-specific anatomical characteristics. For superomedial tumors, the diced ADM with paste-type micronized technique offered superior contour refinement; for inferomedial tumors, the diced ADM with sheet technique provided crucial structural support; and for superolateral tumors, the diced ADM-only technique achieved excellent outcomes with reduced material requirements.

The discrepancy between physician assessments and patient satisfaction, particularly in medial reconstructions, underscores the importance of incorporating patient perspectives in outcome evaluation. These observations provide initial insights that may guide surgeons in optimizing technique selection based on tumor location, although prospective multicenter validation is required before formal recommendations can be established. By tailoring the ADM technique to the specific anatomical challenges of each breast quadrant, surgeons may potentially maximize both objective aesthetic outcomes and subjective patient satisfaction. This personalized approach represents a promising direction in the evolving field of oncoplastic breast surgery that warrants further investigation through prospective, multi-institutional studies to establish more definitive, evidence-based protocols.

## Figures and Tables

**Table 1:** Patient demographics and tumor characteristics.

Characteristic	Diced ADM with Sheet Technique (n = 102)	Diced ADM with Paste-Type Micronized Technique (n = 70)	Diced ADM-Only Technique (n = 57)	Total (n = 229)	*p*-Value ^a^
Age (years) ^b^	49.3 ± 9.2	49.1 ± 9.5	48.8 ± 9.0	49.1 ± 9.2	0.871
BMI (kg/m^2^) ^b^	23.7 ± 3.2	23.8 ± 3.3	23.5 ± 3.1	23.7 ± 3.2	0.795
Tumor location ^c^					0.532
Superomedial	28 (27.5)	20 (28.6)	12 (21.1)	60 (26.2)	
Superolateral	43 (42.2)	29 (41.4)	29 (50.9)	101 (44.1)	
Inferomedial	11 (10.8)	8 (11.4)	4 (7.0)	23 (10.0)	
Inferolateral	20 (19.6)	13 (18.6)	12 (21.1)	45 (19.7)	
Histological type ^c^					0.998
Invasive ductal carcinoma	73 (71.6)	50 (71.4)	41 (71.9)	164 (71.6)	
Ductal carcinoma in situ	23 (22.5)	16 (22.9)	13 (22.8)	52 (22.7)	
Invasive lobular carcinoma	4 (3.9)	3 (4.3)	2 (3.5)	9 (3.9)	
Others	2 (2.0)	1 (1.4)	1 (1.8)	4 (1.7)	
Mean resected weight (g) ^b^	22.6 ± 7.9	20.3 ± 9.6	18.5 ± 7.5	20.8 ± 8.5	0.054
Mean ADM volume (cm^3^) ^b^	25.2 ± 6.6	21.6 ± 4.8 ^d^	19.5 ± 4.5 ^e,f^	22.5 ± 5.8	**<0.001 ** ^a^
Radiotherapy ^c^	100 (98.0%)	70 (100%)	56 (98.2%)	226 (98.7%)	0.972
Chemotherapy ^c^	76 (74.5%)	53 (75.7%)	42 (73.7%)	171 (74.7%)	0.935
Local recurrence ^c^	2 (2.0%)	1 (1.4%)	0 (0%)	3 (1.3%)	0.924
Follow-up completion ^c^	100 (98.0)	68 (97.1)	56 (98.2)	224 (97.8)	0.887

Abbreviations: ADM, acellular dermal matrix; BMI, body mass index. **^a^** *p* < 0.05 considered significant (boldface). ^b^ Mean ± standard deviation. ^c^ Number (percentage). ^d^ *p* = 0.008 vs. diced ADM with sheet technique. ^e^ *p* < 0.001 vs. diced ADM with sheet technique. ^f^ *p* = 0.012 vs. diced ADM with paste-type micronized technique.

**Table 2:** Physician-assessed aesthetic outcomes by tumor location and ADM technique.

ADM Technique	Superomedial (n = 60)	Superolateral (n = 101)	Inferomedial (n = 23)	Inferolateral (n = 45)	*p*-Value ^a^
Diced ADM with sheet technique (n = 102)	3.0 ± 0.6 (n = 28)	3.4 ± 0.5 (n = 43)	2.9 ± 0.7 (n = 11)	3.3 ± 0.5 (n = 20)	**<0.001**
Diced ADM with paste-type micronized technique (n = 70)	3.2 ± 0.5 (n = 20)	3.5 ± 0.4 (n = 29)	3.1 ± 0.6 (n = 8)	3.4 ± 0.5 (n = 13)	**<0.001**
Diced ADM-only technique (n = 57)	2.8 ± 0.6 ^b^ (n = 12)	3.6 ± 0.4 ^c^ (n = 29)	2.7 ± 0.7 ^d^ (n = 4)	3.5 ± 0.4 (n = 12)	**<0.001**
*p*-value ^e^	**0.032**	**0.020**	**0.005**	0.289	

Abbreviation: ADM, acellular dermal matrix. Values are mean ± standard deviation. Aesthetic scale: 1 = poor to 4 = excellent. ^a^ Row comparisons. ^b^ Cohen’s d = 0.71 vs. diced + paste. ^c^ Cohen’s d = 0.44 vs. diced + sheet. ^d^ Cohen’s d = 0.29 vs. diced + sheet. ^e^ Column comparisons. Boldface indicates *p* < 0.05.

**Table 3:** Patient satisfaction scores by tumor location and ADM technique.

ADM Technique	Superomedial (n = 60)	Superolateral (n = 101)	Inferomedial (n = 23)	Inferolateral (n = 45)	*p*-Value ^a^
Diced ADM with sheet technique (n = 102)	2.8 ± 0.7 (n = 28)	3.5 ± 0.4 (n = 43)	2.7 ± 0.7 (n = 11)	3.4 ± 0.5 (n = 20)	<0.001
Diced ADM with paste-type micronized technique (n = 70)	2.9 ± 0.6 (n = 20)	3.6 ± 0.4 (n = 29)	2.8 ± 0.6 (n = 8)	3.5 ± 0.4 (n = 13)	<0.001
Diced ADM-only technique (n = 57)	2.6 ± 0.7 ^b^ (n = 12)	3.7 ± 0.3 (n = 29)	2.5 ± 0.7 ^c^ (n = 4)	3.6 ± 0.4 (n = 12)	<0.001
*p*-value ^d^	**0.023**	0.157	**0.011**	0.312	

Abbreviation: ADM, acellular dermal matrix. Values are mean ± standard deviation. Satisfaction scale: 1 = not satisfied to 4 = very satisfied. ^a^ Row comparisons. ^b^ *p* = 0.023 vs. diced + paste. ^c^ *p* = 0.011 vs. diced + sheet. ^d^ Column comparisons. Boldface indicates *p* < 0.05.

**Table 4:** Complications by tumor location and ADM technique.

Location/Complication Type	Diced ADM with Sheet Technique % (n/N)	Diced ADM with Paste-Type Micronized Technique % (n/N)	Diced ADM-Only Technique % (n/N)	Adjusted OR ^a^ (95% CI)	*p*-Value
Superomedial quadrant					
Depression deformity	6.8 (7/102)	7.1 (5/70)	8.3 (1/12)	1.21 (0.68–2.15)	0.872
Bulging deformity	1.0 (1/102)	0.0 (0/70)	8.3 (1/12)	7.62 (1.29–45.21)	**0.025**
Dermal irregularity	0.0 (0/102)	4.3 (3/70)	8.3 (1/12)	6.94 (1.07–43.71)	**0.042**
Seroma	3.9 (4/102)	0.0 (0/70)	0.0 (0/12)	0.95 (0.89–1.02)	0.582
Hematoma	3.9 (4/102)	7.1 (5/70)	0.0 (0/12)	1.82 (0.48–6.95)	0.488
Superolateral quadrant					
Depression deformity	5.8 (6/102)	4.3 (3/70)	3.5 (2/57)	0.85 (0.52–1.38)	0.758
Bulging deformity	0.0 (0/102)	0.0 (0/70)	0.0 (0/57)	-	-
Dermal irregularity	2.9 (3/102)	1.4 (1/70)	3.5 (2/57)	1.27 (0.57–2.84)	0.482
Seroma	2.9 (3/102)	0.0 (0/70)	0.0 (0/57)	0.97 (0.92–1.01)	0.615
Hematoma	0.0 (0/102)	4.3 (3/70)	1.8 (1/57)	2.41 (0.87–6.64)	0.722
Inferomedial quadrant					
Depression deformity	5.9 (6/102)	8.6 (6/70)	25.0 (1/4)	5.38 (1.19–24.46)	**0.038**
Bulging deformity	1.0 (1/102)	0.0 (0/70)	0.0 (0/4)	0.99 (0.97–1.01)	0.512
Dermal irregularity	0.0 (0/102)	4.3 (3/70)	25.0 (1/4)	6.75 (1.27–35.62)	**0.045**
Seroma	3.9 (4/102)	0.0 (0/70)	0.0 (0/4)	0.96 (0.92–1.00)	0.582
Hematoma	2.9 (3/102)	7.1 (5/70)	0.0 (0/4)	2.44 (0.58–10.32)	0.488
Inferolateral quadrant					
Depression deformity	4.9 (5/102)	4.3 (3/70)	8.3 (1/12)	1.58 (0.78–3.19)	0.568
Bulging deformity	0.0 (0/102)	0.0 (0/70)	0.0 (0/12)	-	-
Dermal irregularity	0.0 (0/102)	0.0 (0/70)	0.0 (0/12)	-	-
Seroma	1.9 (2/102)	0.0 (0/70)	0.0 (0/12)	0.98 (0.95–1.01)	0.725
Hematoma	1.9 (2/102)	4.3 (3/70)	0.0 (0/12)	1.76 (0.39–7.92)	0.495

Abbreviations: ADM, acellular dermal matrix; OR, odds ratio; CI, confidence interval. ^a^ Adjusted for age, BMI, tumor location, and adjuvant therapy; reference: diced + sheet. Boldface indicates *p* < 0.05.

**Table 5:** Comprehensive comparison of ADM techniques: Aesthetic outcomes, patient satisfaction, complications, and economic considerations.

Outcome Parameter	Diced ADM with Sheet Technique (n = 102)	Diced ADM with Paste-Type Micronized Technique (n = 70)	Diced ADM-Only Technique (n = 57)	*p*-Value
Aesthetic outcomes				
Mean physician score	3.2 ± 0.6	3.3 ± 0.5	3.2 ± 0.7	0.652
ICC among evaluators	0.83	0.81	0.82	0.761
Patient satisfaction				
Mean satisfaction score	3.1 ± 0.6	3.2 ± 0.5	3.1 ± 0.7	0.783
Correlation with physician score	0.74	0.79	0.75	0.421
Complications				
Overall complication rate	14.7% (15/102)	15.7% (11/70)	17.5% (10/57)	0.852
Major complications	3.9% (4/102)	4.3% (3/70)	5.3% (3/57)	0.926
Economic considerations				
Mean ADM volume (cm^3^)	25.2 ± 6.6	21.6 ± 4.8	19.5 ± 4.5	**<0.001**
Relative material cost	1.00 (reference)	0.91	0.85	**<0.001**

Abbreviations: ADM, acellular dermal matrix; ICC, intraclass correlation coefficient. Values are presented as mean ± standard deviation or percentage (number). Boldface indicates *p* < 0.05.

## Data Availability

The data presented in this study are available upon reasonable request from the corresponding author. The data are not publicly available due to privacy restrictions and institutional policies regarding clinical data protection, in accordance with the principles outlined in the Declaration of Helsinki.
